# Experiences of orthopaedic patients referred from regional hospitals to an intermediate hospital in Namibia: A qualitative study

**DOI:** 10.4102/curationis.v49i1.2772

**Published:** 2026-04-02

**Authors:** Lydia Witbeen, Emma M. Nghitanwa, Tuwilika Endjala

**Affiliations:** 1 School of Nursing and Public Health, Faculty of Health Sciences and Veterinary Medicine, University of Namibia, Windhoek, Namibia; 2 Department of Community and Mental Health, Faculty of Health Sciences and Veterinary Medicine, University of Namibia, Windhoek, Namibia

**Keywords:** experience, orthopaedic, patients, intermediate hospital, referral

## Abstract

**Background:**

The effectiveness of primary and special healthcare systems depends on the patient referral system. In Namibia, most patients that require specialised care are referred to a referral hospital in Windhoek because of inadequate facilities, equipment and health workers in most of the regions.

**Objectives:**

This study aimed to investigate the experiences of orthopaedic patients referred from regional hospitals to Katutura Intermediate Hospital in Namibia. The study was conducted at the Katutura Intermediate Hospital in Namibia.

**Method:**

A qualitative approach with descriptive-exploratory design was utilised. The target population comprised of 50 patients referred to the orthopaedic department from the regions to Katutura Intermediate Hospital in Namibia, and purposive sampling was used to sample participants. Data were collected among 28 participants till there was no more new information emerging. Data were collected through semi-structured interviews and analysed using thematic analysis.

**Results:**

The study found that patients had varied experiences related to healthcare provision, with positive and negative experiences reported. Most patients were satisfied with the quality of healthcare services provided, while some indicated negative experiences. Areas for improvement in the referral transportation experience were identified, with a need for better vehicles and improved communication.

**Conclusion:**

The study reveals positive and negative experiences among participants, but most participants have positive experiences in service deliveries during hospital referrals.

**Contribution:**

The study contributed to the literature by revealing the experiences of patients referred that might assist the management to develop strategies to address the challenges identified for quality healthcare.

## Introduction

Healthcare systems of most countries in Southern Africa are structured for patients to seek healthcare or treatment at the primary healthcare facilities, and they can be referred to the secondary healthcare facilities if necessary (Matolengwe, Murray & Okafor 2024). The collaboration of primary and intermediate healthcare facilities results in the patient referral system that is observed in almost every nation of the world. According to Seyed-Nezhad, Ahmadi and Akbari-Sari (2021), for a patient referral system to be effective, it is essential for the healthcare workers to take accountability for the continuousness of the healthcare to patients, and for the work processes in the patient referral system to be systematised. According to Coombs, Campbell and Caringi (2022), improving access to specialty care has been recognised as a challenge in the distribution of healthcare services, particularly given a cumulative burden of enduring diseases. Hence, identifying and tackling problems that affect access to quality healthcare for patients referred to speciality healthcare for non-emergent dealings and how these deficiencies can be accomplished through healthcare system transfer involvements is significant to improve care for patients with deadly conditions. The effectiveness of primary and special healthcare systems depends on the patient referral system (Seyed-Nezhad et al. 2021). There are numerous reasons as to why a patient might be referred to a regional hospital for care such as for diagnosis, management guidance, and cure beyond the scope of the primary healthcare facility. Therefore, with the growing plea for specialty care, and limited resources to meet the demand, patient referral quality is progressively important to safeguard efficient patient flow across the primary to specialty care interface. Hence, patient referrals must be required, appropriate, well-timed, and well communicated.

In 2022, there were about 2000 patients that were treated at the state orthopaedic department, and about 60% of those patients were treated at Katutura Intermediate Hospital. As healthcare professionals, the authors witnessed instances whereby patients are referred to Windhoek from Katima Mulilo, which is 1325 km away, or Rundu to Windhoek, which is 715 km away. Patients coming from different regions to Windhoek with patient buses for admission sometimes still need to wait for hours at the outpatient department to be assisted and admitted to specific wards. Katutura Intermediate Hospital has a bed capacity of 120 beds. However, if the number of referrals exceeds 100 per month and ± 30 per week, it results in a massive challenge in terms of bed capacity above its capacity. Katutura Intermediate Hospital is already operating above its capacity, and adding the number of referral patients will result in far-reaching ramifications on both the patients and staff. The authors could not find any study that investigated the view of referring patients to the orthopaedic department at the study site.

### Study purpose and objectives

The study’s aim was to investigate experiences of patients referred from regional hospitals to Katutura Intermediate Hospital in Namibia. The study objectives were to explore and describe the experiences of patients during hospital referral to Katutura Intermediate Hospital in Namibia and to identify the contributory factors to the experiences experienced by patients during hospital referral to Katutura Intermediate Hospital.

## Research methods and design

The study employed a qualitative approach of descriptive, exploratory design.

### Study setting

The study took place in Katutura Intermediate Hospital in Khomas region in Namibia. The Intermediate Hospital is the largest hospital in Namibia with a bed occupancy of 886. It receives an average of ± 50 patients per month and ± 10 patients on a weekly basis from different hospitals from 13 different regions in the orthopaedic department. There are three wards for orthopaedic patients consisting of 120 beds (Ministry of Health and Social Services [MoHSS] 2022). Patients were identified by ward staff and were approached by authors to be invited to participate in the study. Inclusion criteria included being a current inpatient referred to orthopaedic ward at Katutura Intermediate Hospital from other regions and patients aged between 18 years and 65 years who have been admitted pin Katutura Intermediate Hospital for a minimum of 5 days. A purposive sampling technique was used to select the sample of participants who can provide rich information for the study. The population of the study consisted of all orthopaedic patients referred to Katutura Intermediate Hospital.

### Data collection and tools

Data were collected by authors with a semi-structured interview guide developed by authors in English guided by literature review. The data collection tool was piloted before the main study among five patients with similar characteristics to the participants for the main study, and they were not included in the main study. No changes were made after piloting. A semi-structured interview tool consisted of two sections; section A concentrated on socio-demographic data, and section B focused on the experiences of patients during hospital referral to Katutura Intermediate Hospital. The central question was ‘tell me about your experience during hospital referral to Katutura Intermediate Hospital’. Data were recorded with a voice recorder, and field notes were also used during data collection.

Data were collected for a period of 1 month by conducting interviews in a private quiet room at the hospital. Interviews were conducted during the time when the patients were not engaged with care by healthcare providers. The total number of people interviewed were 28, and data saturation was reached, meaning there was no new information emerged, or when provided, information was re-emerging; that indicated that no new themes would occur. The semi-structured interviews were recorded using a voice recorder, and field notes were taken. Each interview lasted for 30–35 min. To ensure anonymity in the study, participants were identified with codes.

### Data analysis

To analyse primary data from the semi-structured interviews, the study used thematic analysis. Once the interviews were completed, the authors transcribed audio recordings. The authors typed out the audio recordings word for word (verbatim) as mentioned by the participants. The data were presented in illustrative quotes, themes with analysis and interpretation of the themes.

### Trustworthiness

To ensure trustworthiness, credibility, transferability, dependability and confirmability were addressed. In-depth face-to-face interviews were recorded, and nonverbal responses were noted in the field note. During the interview process, repetition and paraphrasing were done with the participants to ensure credibility. To ensure transferability, non-probability sampling was used to maximise different experiences and perceptions of individuals. All data were recorded, written, analysed and constructed into main themes and sub-themes for conformability. Raw data were coded, and an audit trail was done of all phrases under the supervision of the research supervisors. A pilot study was conducted in a tertiary hospital in the same country among five orthopaedic patients referred, and no changes were made in the data collection tool.

### Ethical considerations

Ethical clearance to conduct this study was obtained from the University of Namibia School of Nursing Ethical Committee (reference number: SoNPH/2023). The authorisation letter to conduct the study was obtained from the MoHSS research ethics committee and from the management of the Intermediate Hospital (reference number: 20042023). Informed consent was obtained from each participant after a full disclosure of the research was given. This consent form stated that the respondents have the right to withdraw from the study at any time without penalty. To ensure anonymity, the authors allocated codes to every respondent instead of using real names. Interview transcripts were kept safe and only accessible to the authors. Questions that may trigger any form of psychological or physical harm to the participants were avoided. Arrangements were made with the social work in Katutura Intermediate Hospital to refer participants that may show the signs of distress during data collection. The authors acted for the benefit of the patient. Participants were chosen based on research objectives instead of investigators’ interest. Inclusion and exclusion criteria were structured based on the research protocol which was adhered to so that all subjects in the study population had equal opportunities to participate.

## Results

### Characteristics of participants

Data were collected from 28 participants based on data saturation. Five (18%) were male, and 23 (82%) were female. Their ages ranged from 19 years to 52 years of age. Regarding marital status, 7(25%) were single, 9 (32%) were married, 6 (21%) were separated, and cohabitating respectively. Results also show that 7 (25%) participants were employed, 12 (43%) were unemployed and 9 (32%) were self-employed.

### Results on patient experiences regarding referrals

Three themes and 6 sub-themes emerged from the data, as displayed in Table 1.

**TABLE 1:** Themes and subthemes regarding patients’ experiences.

Main theme	Sub-theme
1. Patient feelings and experiences	1.1. Feelings and thoughts about referral to an intermediate hospital 1.2. Visitation and support from family and friends 1.3. Experience in the orthopaedic department of the Intermediate Hospital
2. Evaluation of healthcare services	2.1. Perception on health services at Intermediate Hospital
3. Recommendations for improvement	3.1. Interventions by the Ministry of Health and Social Services (MoHSS) to mitigate negative experiences during hospital referral to Intermediate Hospital 3.2. Suggestions for improving referral transportation experience from regions to Intermediate Hospital

MoHSS, Ministry of Health and Social Services.

#### Theme 1: Patient feelings and experiences

**Sub-theme 1.1: Feelings and thoughts about referral to the Intermediate Hospital:** Participants express different thoughts about being referred such as discomfort about the journey to the intermediate hospital in other region; however, some participants also felt that the specialised healthcare services they received made it worth the trip as quoted by some participants:

‘The journey from my region was quite long and exhausting, but I was happy to be referred to the Intermediate Hospital because it provided me with access to more specialized healthcare services.’ (Participant 6, female, 33 years old)

Another had a similar experience, stating:

‘The journey from my region to Intermediate Hospital was quite long and tiring but it was worth it for the excellent medical care I received at the orthopaedic department.’ (Participant 7, male, 52 years old)

Some participants had very positive experiences, complimenting the staff and facilities, while others found their experiences to be frustrating because of long wait times and unclear communication about treatment plans.

One participant had a positive experience, stating:

‘My experience at the orthopaedic department at the Intermediate Hospital was quite good. The doctors and nurses were very friendly and accommodating.’ (Participant 1, male, 26 years old)

Another also stated that:

‘I was initially apprehensive about being referred to Intermediate Hospital, but my experience there completely changed my perspective. The staff were extremely professional, friendly, and knowledgeable. They made me feel comfortable and at ease throughout my entire visit. The facilities were clean and well-maintained, and I received top-notch medical care. I left feeling grateful for the compassionate and expert care I received at Intermediate Hospital.’ (Participant 3, female, 30 years old)

However, one had a more frustrating experience, saying:

‘I have had a somewhat frustrating experience at the orthopaedic department at the Intermediate Hospital. Although the staff are friendly and professional, the wait times have been very long and communication regarding the treatment plan has been unclear at times.’ (Participant 4, female, 48 years old)

Another also stated that:

‘I had a terrible experience being referred to Intermediate Hospital. The entire process was a nightmare, and I felt like I was just another number on their list, rather than a human being. The staff there seemed completely indifferent to my needs, and I was left waiting for hours on end without any updates or information. The facilities were also extremely run down, and I felt very uncomfortable and unsafe during my stay. Overall, I would never recommend this hospital to anyone, and I would be extremely hesitant to ever go back myself.’ (Participant 5, female, 32 years old)

Also, when asked about their thoughts and feelings upon referral to Intermediate Hospital, participants had varied responses. Some were nervous about the quality of care they would receive but felt better after receiving information prior to their visit:

‘At first, I was a bit apprehensive about being transferred to Intermediate Hospital because I had never been to the capital city before. However, the healthcare professionals who referred me provided me with adequate information about the hospital and what I could expect.’ (Participant 11, male, 40 years old)

Others were more positively curious about their visit:

‘My first thoughts when I heard that I was being transferred to the Intermediate Hospital were ones of curiosity and intrigue. I had never been to the hospital before and was interested to see what it would be like.’ (Participant 15, female, 19 years old)

Overall, the responses provided insight into the varied patient experiences and emotions related to healthcare provision.

**Sub-theme 1.2: Visitation and support from family and friends:** Under the sub-theme of ‘Visitation and support from family and friends’, participants had varied experiences depending on the presence or absence of loved ones in the capital city where the hospital is. Participants who had relatives and friends in the city appreciated their company and support during their stay at the hospital:

‘Their visits were a great source of comfort, and it helped me to deal with the stress and anxiety that I was feeling. I really appreciate their support during that difficult time.’ (Participant 16, female, 38 years old)

One shared a similar experience, saying:

‘It’s always great to see familiar faces and catch up on what’s been happening outside of the hospital walls.’ (Participant 23, male, 25 years old)

However, some participants did not have any relatives or friends in the city where the hospital is situated to visit them, and they expressed feelings of isolation:

‘It can be tough being so far away from loved ones, but I’m grateful for the support of the hospital staff and my fellow patients.’ (Participants 12, female, 42 years old)

Another shared a similar experience, saying:

‘It can be tough feeling isolated in a new environment, but the hospital staff have been incredibly supportive and have helped me settle in.’ (Participants 25, female, 52 years old)

Overall, the responses highlighted the importance of emotional support from loved ones during hospital stays and the impact it can have on patients’ well-being.

**Sub-theme 1.3: Experience in the orthopaedic department of Intermediate Hospital:** Most participants had a positive experience at the orthopaedic department, praising the staff and facilities:

‘The staff was very attentive, and they kept me informed about my treatment plan. I found the facilities and equipment to be adequate, and I felt that I was receiving the best care possible.’ (Participant 1, male, 26 years old)

Another also had a similar experience and said:

‘The medical staff are highly competent and provide excellent care, and the facilities are clean and well-maintained.’ (Participant 11, male, 40 years old)

On the other hand, Participant 10 had a mixed experience, highlighting that though the medical care and treatment received were good, they had negative experiences with certain staff members. However, Participant 13 had an excellent experience and praised the staff’s skill, knowledge and kindness.

#### Theme 2: Evaluation of healthcare services

**Sub-theme 2.1: Perception of health services at Intermediate Hospital:** Results show that almost all participants are satisfied with the quality of healthcare services provided by the Intermediate Hospital. Participant 14 describes the hospital’s services as ‘excellent’, with compassionate and attentive medical staff who work hard to ensure that patients receive high-quality care that meets their individual needs and preferences. This highlights the importance of patient-centredness in healthcare service delivery.

One participant describes the medical staff as:

‘… knowledgeable, skilled, and efficient, and they go out of their way to ensure that patients are comfortable and well-informed about their treatment.’ (Participant 21, female, 33 years old)

This statement shows that the medical professionals take an active role in providing patient education, thereby contributing to the empowerment of patients with knowledge and information regarding their healthcare.

Interestingly, Participant 5 highlights the Intermediate Hospital as a vital healthcare facility that provides quality medical care to patients who might not have easy access to other healthcare facilities. This shows that the hospital plays a significant role in improving healthcare access and reducing healthcare disparities within the community.

#### Theme 3: Recommendations for improvement

**Sub-theme 3.1: Interventions by the Ministry of Health and Social Services to mitigate negative experiences during hospital referral to the Intermediate Hospital:** Participants suggest that there are several interventions that MoHSS could implement to mitigate negative experiences during hospital referral to the Intermediate Hospital. These interventions range from information provision to improving transportation to providing more amenities and medical personnel.

One participant’s recommendation of providing adequate support before, during and after referral could be very beneficial for patients as they often experience anxiety and uncertainty during hospital referrals as quoted below:

‘It would be helpful if the MoHSS could collaborate with community healthcare workers to provide follow-up care and support to patients once they return to their communities.’ (Participant 21, female, 33 years old)

This follow-up care would not only provide patients with increased support but would also ensure their adherence to treatment.

Furthermore, the call for improving transportation is not new, and another proposes that:

‘MoHSS could provide alternative modes of transport, such as shuttle buses.’ (Participant 28, female, 40 years old)

This could make it easier for patients to access healthcare services at the hospital, especially those who live far from the facility. This measure could also reduce the burden on the few ambulances available, thereby reducing the waiting time for patients.

Additionally, Participant 4’s suggestion of providing more amenities and support services within the hospital could help reduce the anxiety patients experience during hospital referrals:

‘MoHSS could consider providing more amenities and support services within the hospital, such as creating comfortable waiting areas for patients and their families or providing access to counselling or other mental health services.’ (Participant 4, female, 48 years old)

These services could provide patients with more comfort and support during their hospital visits, thereby increasing their satisfaction with the healthcare experience.

It is clear that there are several interventions that MoHSS could implement to mitigate negative experiences during hospital referral to Intermediate Hospital. These interventions focus on enhancing the comfort and support provided to patients and could significantly improve patient experience and satisfaction with healthcare services. As one participant puts it:

‘Increasing the number of medical personnel available at the Intermediate Hospital would make the hospital experience more pleasant for everyone involved.’ (Participant 5, Female, 32 years old)

**Sub-theme 3.2: Suggestions for improving referral transportation experience from the regions and town to the Intermediate Hospital:** The responses suggest that there are several areas for improvement in the referral transportation experience from regions to the Intermediate Hospital, ranging from better vehicles to improved communication.

One participant’s feedback on the cramped and uncomfortable vehicle highlights a key area for improvement in transportation as quoted:

‘The vehicle was cramped and uncomfortable, and the journey was long and tiring.’ (Participant 1, Male, 26 years old)

This feedback suggests that there is a need for vehicles that are more spacious and comfortable.

Additionally, another participant’s feedback on the poor condition of the vehicle is also important to note:

‘The journey was quite long and bumpy, and the vehicle that transported me was in poor condition.’ (Participant 22, female, 20 years old)

This feedback suggests that there is a need for a more rigorous maintenance schedule for vehicles used in referral transportation.

Participants 9 and 11’s positive feedback on their transportation experiences highlights the importance of clear communication and transparency during the transportation process:

‘I was given information about the transport schedule and what I could expect during the journey.’ (Participant 9, female, 28 years old)

This feedback suggests that patients have a need for information on transportation schedules and expectations to help them prepare for the journey. Participant 11’s initial apprehension about transportation also suggests a need for clear communication on what patients can expect during their referral transportation to help reduce anxiety and uncertainty.

## Discussion

The demographic data show that the study had a slightly greater representation of female patients compared to male patients. The age range of the patients was broad, with the youngest respondent aged 19 years and the older respondent being 52 years old. This suggests that patients of different age groups are referred to Katutura Intermediate Hospital for specialised healthcare services. Nolan, Kuhner and Dy (2019) had similar demographic findings in their study.

Regarding the participants’ marital status, the study found that an almost equal number of respondents were single, married, separated, or cohabitating. This suggests that marital status might not have a significant effect on the referral experience to the Intermediate Hospital, and patients of all marital statuses might experience similar challenges. In terms of employment status, the data show that there were more unemployed participants, followed by self-employed. This finding is not surprising, as unemployed and self-employed individuals might face more financial constraints when accessing healthcare services and might also face more challenges in terms of transportation and support during the referral process. Some patients expressed discomfort about the journey to the referral hospital, but most felt that the specialised healthcare services they received made it worth the trip. Ringberg and Fleten (2018) contradict the findings above as they found that 65% of unwanted appointments were avoided resulting in shorter waiting time for genuine patient, higher patient satisfaction and more patients being attended ultimately in Windhoek. Additionally, participants had mixed views about their experiences at the orthopaedic department. Some participants had very positive experiences, complimenting the staff and facilities, while others found their experiences to be frustrating because of long wait times and unclear communication about treatment plans. These findings are similar to those of Mselle et al. (2021), who found that in Tanzania, patients mostly experienced the unavailability of hospital transport as well as the lack of a reliable feedback mechanism in the state’s patient referral system. In addition, some patients were nervous about the quality of care they would receive but felt better after receiving information prior to their visit. These results reflect the findings of Winpenny et al. (2016), who found that the pressure to improve healthcare services delivery is increasing in order to respond to the cumulative prevalence of chronic conditions. As a result, advanced methods for the delivery of healthcare have been projected, emphasising improved integration between primary and specialty care. Overall, the findings provided insight into the varied patient experiences and emotions related to healthcare provision. The three wards of orthopaedic department in Katutura Intermediate Hospital have a bed capacity of 120 beds. However, if the number of referrals exceeds 100 per month and ± 30 per week, it results in a massive challenge in terms of bed capacity. Overall, these responses provide insight into the patients’ experiences at the orthopaedic department and emphasise the importance of providing patients with good medical care and treating them with respect and kindness to help them have a positive hospital experience.

The second sub-theme of theme 1: Visitation and support from family and friends, revealed that participants had varied experiences depending on the presence or absence of loved ones in Windhoek. Participants who had relatives and friends in the city appreciated their company and support during their stay at the hospital. However, some participants did not have any relatives or friends in Windhoek to visit them, and they expressed feelings isolated. Overall, the results highlighted the importance of emotional support from loved ones during hospital stays, and the impact it can have on patients’ well-being.

The third sub-theme of theme 1: Experience in the orthopaedic department of the Intermediate Hospital revealed that participants had varied experiences. Most participants had positive experience, praising the staff and facilities. On the other hand, some participants had a mixed experience, highlighting that though the medical care and treatment received were good, they had negative experiences with certain staff members. These findings may support the report by the MoHSS Report (2022) that appreciates the important role played by the orthopaedic physicians at state facilities as they provide services to the Namibian people, as 90% of the people they treat do not have medical aid. However, other participants had excellent experience and praised the staff’s skill, knowledge, and kindness. Their positive experiences may indicate that the hospital is offering good services to patients. Overall, these findings provide insight into the patients’ experiences at the orthopaedic department and emphasise the importance of providing patients with good medical care and treating them with respect and kindness to help them have a positive hospital experience. Similarly, Kwame and Petrucka (2021) alluded the importance of quality healthcare provision including patients and family respect and support for positive experience and positive patients’ outcomes. All participants indicated that they were satisfied with the quality of healthcare services provided by the Intermediate Hospital. This highlights the importance of patient-centredness in healthcare service delivery. Moreover, some participants highlighted that Katutura Intermediate Hospital is a vital healthcare facility that provides quality medical care to patients who might not have easy access to other healthcare facilities. This shows that the hospital plays a significant role in improving healthcare access and reducing healthcare disparities within the community. The responses show that the healthcare professionals at Intermediate Hospital are knowledgeable, skilled, efficient, compassionate and patient-centred. The hospital’s facilities and equipment were also satisfactory, contributing to the provision of high-quality health services to its patients. Additionally, Maseke and Nyathi (2021) also revealed that healthcare professionals at the intermediate hospital are very skilled.

The study findings revealed that there are several interventions that the MoHSS could implement to mitigate negative experiences during hospital referral to the Intermediate Hospital. These interventions range from information provision to improving transportation to providing more amenities and medical personnel. Furthermore, the study found that some participants recommended that MoHSS should provide adequate support before, during, and after referral, which could be very beneficial for patients as they often experience anxiety and uncertainty during hospital referrals. Furthermore, the call for improving transportation could make it easier for patients to access healthcare services at the hospital, especially those who live far from the health facility. This measure could also reduce the burden on the few ambulances available, thereby reducing the waiting time for patients. Again, there are several interventions that MoHSS could implement to mitigate negative experiences during hospital referral to the Intermediate Hospital. These interventions focus on enhancing the comfort and support provided to patients and could significantly improve patient experience and satisfaction with healthcare services. Tjitemisa (2017) reported the same recommendations because the referral hospitals were stretched beyond limits.

Furthermore, some participants’ feedback on the cramped and uncomfortable vehicle highlights a key area for improvement in transportation. The results indicated a need for vehicles that are more spacious and comfortable. The study results highlight a need for improvement in various aspects of the referral transportation experience. From better vehicles to improved communication, these areas for improvement could significantly enhance the patient experience and reduce the stress associated with referrals to the Intermediate Hospital. Participant 2’s feedback on their surprisingly good transportation experience could be an indication that some elements are working well, and that they could be scaled for wider use. The responses highlight a need for improvement in various aspects of the referral transportation experience. From better vehicles to improved communication, these areas for improvement could significantly enhance the patient experience and reduce the stress associated with referrals to the Intermediate Hospital. Participants recommended improvement of transportation to providing more amenities and medical personnel. Mselle, Sirili Anaeli and Massawe (2021) made similar recommendations about referral procedures in the rural and semi-urban district hospitals in Tanzania. The recommendations provided suggest that there is room for improvement in the referral process, and such improvements could significantly enhance the patient experience and satisfaction with healthcare services.

### Limitations

The study was limited to patients referred to the Intermediate Hospital; therefore, the results cannot be generalised. Furthermore, there was anticipated that respondents were reluctant to give accurate responses because of confidentiality reasons that might lead to bias. However, the researcher explained the main purpose of the study and the anonymity of the respondents before they participated in the study.

## Conclusion

The study found a slightly higher representation of female patients with a broad age range. Employment status, however, revealed that more unemployed and self-employed patients were referred.

The study revealed that participants expressed discomfort with the journey to the hospital, but the specialised healthcare services made it worthwhile. Patients had mixed experiences at the orthopaedic department, and emotional support from loved ones was found to be important. Some patients had negative experiences with staff, but overall good medical care and treating patients with respect and kindness are important for a positive hospital experience. Overall, the findings highlighted the importance of providing patients with good medical care and treating them with respect and kindness to help them have a positive hospital experience. It also emphasised the need for improved communication and integration between primary and specialty care to enhance healthcare services delivery.

Additionally, the study found that the Intermediate Hospital is making efforts to provide quality healthcare services to its patients, but there is still room for improvement, especially in reducing waiting times. The need for interventions to enhance patient comfort and support during referrals was highlighted, such as improving transportation, providing more amenities and medical personnel. Better vehicles and improved communication are needed for referral transportation experiences. It is recommended that there is a need for policy makers in the MoHSS to focus on providing more patient-centred care to ensure a positive experience and to meet patients’ healthcare needs. Furthermore, it is recommended that the MoHSS to improve transportation in terms of providing well-maintained vehicles and avoiding overcrowding in the vehicle to prevent patients’ discomfort.
